# 

**Published:** 1919-01-25

**Authors:** 


### Joint War Committee Nurses.

### DEMOBILISATION PAYMENT SCHEME.

It is important to all trained nurses and others who work under the British Red Cross Society and the Order of St. John of Jerusalem in England that they should clearly get in their mind particulars of the Demobilisation Payment Scheme of the Joint War Committee. We have, therefore, the pleasure to publish this week the following information:

The scheme will not apply to persons whose contracts have been terminated before November 29, 1918.

Persons themselves determining their contracts will receive no special payment.

The scale of payments under this scheme to persons whose contracts are determined by the Committee is based on length of service prior to March 31, 1919, and is as follows:

Scale of Payments.

| | | | | | | | |
|---|---|---|---|---|---|---|---|
| Under 6 months' service | | ... | ... | | Gratuity of | 4 | weeks' pay |
| Over 6 | ,, | but under | 12 | months | ,, | 6 | ,, |
| ,, 12 | ,, | ,, | 18 | ,, | ,, | 8 | ,, |
| ,, 18 | ,, | ,, | 24 | ,, | ,, | 10 | ,, |
| ,, 24 | ,, | ,, | 30 | ,, | ,, | 12 | ,, |
| ,, 30 | ,, | ,, | 36 | ,, | ,, | 14 | ,, |
| ,, 36 | ,, | ,, | 42 | ,, | ,, | 16 | ,, |
| ,, 42 | ,, | ,, | 48 | ,, | ,, | 18 | ,, |
| ,, 48 | ,, | ,, | 54 | ,, | ,, | 20 | ,, |

*Note.*—Payments under the above scheme do not interfere in any way with the payment of Bonus of £7 10s. or Christmas Gift in the ordinary course.

### THE COLLEGE OF NURSING
### (Limited by Guarantee).

CAMBRIDGE CENTRE.

Thursday, January 30.—Professor Nuttall, F.R.S., will give an address at the 1st Eastern General Hospital at 5.30 p.m. on "Some Infectious Diseases that have Attacked our Soldiers." The lecture is open to members of the College and others. Admission to non-members, 6d.

LIVERPOOL CENTRE.

The fourth post-graduate lecture of the session, "Asepsis in Wound Treatment," was given by W. Thelwall-Thomas, Esq., Ch.M., M.R.C.S., on January 15. The lecture, which was illustrated by lantern slides, was greatly appreciated by the nearly 300 nurses who were present.

### Some Hospitals' Gazettes.

### Famine in Babies.

The decrease in the birth-rate, according to *St. Mary's Hospital Gazette*, has been so marked "that even Queen Charlotte's Hospital has scarcely enough cases to supply the needs of its own students." Against this, however, must be put the fact that the students at Queen Charlotte's have probably trebled in number within the last three years owing mainly to the closing up of hospital districts because of war conditions.

### London Hospital War Memorial.

Suggestions are invited by the London Hospital as to the form a memorial to those connected with the hospital and college who have died in the war should take. It is assumed as a matter of course that the names of the fallen shall be inscribed on a tablet or memorial to be placed in one of the buildings or in the garden. But apart from that the idea that is germinating and soon will be waiting for practical expression is the institution of something which "will weave the memory of their sacrifice into the work and life of our college and hospital for all time to come."

### Women Students in Hospitals

The policy of the London Hospital and College in admitting women students is reviewed with satisfaction by the *London Hospital Gazette*. At the present time there are a dozen women students in the college and hospital. The innovation has, it is stated, been "universally popular," and there is no sign so far of the admission affecting adversely the entry of men students. The policy of the "London" in admitting women was, it is added, "speedily followed by University College Hospital and King's College Hospital, so that the schools in London which still close their doors to women are now reduced to four—St. Bartholomew's, St. Thomas's, Guy's, and the Middlesex."

### GIFTS AND BEQUESTS.

The King has forwarded to the Great Northern Central Hospital a cheque for £10, as a special Christmas donation to the funds. From the League of the Roses (per the chairman, Miss M. F. Roby) a further sum of £100, bringing the Leagues contributions for the Women's Ward to £900 during the past nine months.

Mrs. E. G. Rowley, of Gawthorpe Ossett, Yorks, left £250 to the London Temperance Hospital, £200 to the Hospital for Incurable Diseases and some other charities, and varying sums to other institutions.

Brighton charities benefit under the will of Mrs. Mary Hallett, of Hove, who left £500 each to the Brighton, Hove, and Sussex Throat and Ear Hospital, the Brighton and Hove Hospital, and the Brighton and Hove and Preston Dispensary, and the residue, after the lives of her sisters, to the Royal Sussex County Hospital, to be known as the "William Henry and Mary Hallett" Fund.

Under the will of the late Miss Florence Blake, of Weston-super-Mare, the following sums are bequeathed: £3,000 to Muller's Orphanage, Ashley Down, and £2,000 to the Mildmay Mission to the Jews.

Mr. A. O. Worthington, of Maple Hayes, near Lichfield, left an estate valued at £1,356,975; he bequeathed £500 to Burton Infirmary, and a number of smaller legacies to other societies or institutions, including £100 each to the Staffs General Infirmary, the Hammerwich Hospital, and the Royal Bath Hospital, Harrogate.

Alderman Albert Johnson, of Stockport, has left an estate with net personalty of £34,494, the bulk of which goes in equal shares to the Stockport Infirmary and the Stockport Sick Poor and Private Nursing Fund.

Sir George Benjamin Hingley, Bart., left £500 each to the Guest Hospital, Dudley, the Birmingham Eye Hospital, the Knightwick Sanatorium, and the Home for the Dying, Clapham Common.

The trustees of the fund left by the late Mr. G. F. Muntz for the medical charities of Birmingham have made the following grants: £200 to the Queen's Hospital, £70 to the Moseley Hall Convalescent Home for Children, and smaller sums to other Birmingham charities.

Mrs. Hannah Derbyshire, of Oldham, left £1,000 to Oldham Royal Infirmary, £500 to the National Children's Hospital, Bonner Road, and the residue of her estate, subject to some other legacies for charity, her husband would have approved of, partly to be used for the endowment of a playground and recreation institute for children at Lees.

Lieutenant Geoffrey Hughes, of the Grenadier Guards, left £1,000 each to St. Dunstan's Hostel for Blinded Soldiers, the Officers' Families Fund, the Liverpool Workshops and Homes for the Blind, the Royal Southern Hospital, and the Aged Merchant Seamen's Fund; £500 to the Royal Southern Hospital; and £100 each to the Liverpool Home for Incurables and the Liverpool Consumption Hospital and Delamere Sanatorium.

### PASSING EVENTS AND TOPICS.

### Private Auxiliary Hospitals Closing.

All the private auxiliary hospitals are expected to close in the near future. On March 1 the Hospital for Officers at 24 Park Street, London, will discontinue operations; the beautiful house which Mr. Granville Farquhar several years ago lent to the Westminster Division of the B.R.C.S. will revert to the use of its generous owner. Similarly, some time next month, Viscountess Ridley's officers' hospital at her house in Carlton House Terrace will cease to be. This popular hospital has been maintained since 1914, though the Red Cross Society took it over a couple of years ago. The Duchess of Sutherland's naval hospital at her house in Portland Square has just closed after an existence of six months. The closing of these and other hospitals means the release of a large number of V.A.D.s.

### Care Committee Winding Up.

The R.A.M.C. Prisoners of War Care Committee is winding up its affairs, the majority of R.A.M.C. prisoners having been repatriated. In addition to help received from relations, friends, and employers, officers and men of the R.A.M.C. have subscribed £21,000 to the fund, which has been administered in the Officers' Mess, Royal Army Medical College, Grosvenor Road, London.

### Medals for Brave Nurses.

For courage and presence of mind on the occasion of a fire at Oaklands Hospital, Clevedon, the Medal of the Order of the British Empire has been conferred on Catherine Waddell and Gladys Westrope, both nurses. A similar honour has been paid Freda Bowring, also a nurse (institution not stated), who has frequently given skin for grafting on to patients.

### Hospital Interim Report.

Ancoats Hospital, Manchester, has the useful idea of issuing during the year a statement of statistics and financial results. During the first nine months of 1918 the income has increased by nearly £2,000 and the expenditure by £1,300, as compared with the same period in 1917. Although the weights of foods consumed, taken generally, have, under the rationing scheme, been greatly reduced, the gross amount of money spent under the heading of provisions remains about the same. Six hundred and forty-seven chickens were used in 1917, and four only in 1918. Fish has increased in consumption from 2,935 lb. to 5,065 lb.

### In a Few Lines.

The suggestion is made that the body of Nurse Cavell, together with that of Capt. Fryatt, should be brought to England on the *Vindictive* when that famous old warship is once more afloat.

"No further enrolments will be made in the General Service Section, Voluntary Aid Detachment, without specific War Office authority," is the announcement made in an Army Council Instruction.

### Forthcoming Events.

The Tuberculosis Society.—Conference of Tuberculosis Officers, on Saturday, January 25, 7 p.m., at the Royal Society of Medicine, 1 Wimpole Street, W., to discuss the status of the Tuberculosis Service under forthcoming legislation. A meeting of the Society will be held on Monday, January 27, at 8.30 p.m., at the Royal Society of Medicine, 1 Wimpole Street, W., when Dr. Dundas Grant will speak on "Tuberculosis in relation to the Upper Air Food Passages."

